# Effects of empagliflozin and target-organ damage in a novel rodent model of heart failure induced by combined hypertension and diabetes

**DOI:** 10.1038/s41598-020-70708-5

**Published:** 2020-08-20

**Authors:** Kristin Kräker, Florian Herse, Michaela Golic, Nadine Reichhart, Sergio Crespo-Garcia, Olaf Strauß, Jana Grune, Ulrich Kintscher, Manal Ebrahim, Michael Bader, Natalia Alenina, Arnd Heuser, Friedrich C. Luft, Dominik N. Müller, Ralf Dechend, Nadine Haase

**Affiliations:** 1 Experimental and Clinical Research Center, Lindenberger Weg 80, 13125 Berlin, Germany; 2 Berlin Institute of Health (BIH), Berlin, Germany; 3 Max-Delbrück Center for Molecular Medicine in the Helmholtz Association, Berlin, Germany; 4 DZHK (German Centre for Cardiovascular Research), Berlin, Germany; 5 Charité – Universitätsmedizin Berlin, Berlin, Germany; 6 Experimental Ophthalmology, Department of Ophthalmology, Charité – Universitätsmedizin Berlin, Berlin, Germany; 7 Department of Biochemistry, Université de Montréal, Montreal, Canada; 8 Institute of Physiology, Charité – Universitätsmedizin Berlin, Berlin, Germany; 9 Institute of Pharmacology, Center for Cardiovascular Research, Charité – Universitätsmedizin Berlin, Berlin, Germany; 10 Center for Systems Biology, Massachusetts General Hospital, Harvard Medical School, Boston, USA; 11 Technische Universität Berlin, Berlin, Germany; 12 HELIOS-Klinikum, Berlin, Germany

**Keywords:** Cardiology, Experimental models of disease, Translational research, Cardiovascular diseases, Endocrine system and metabolic diseases

## Abstract

Type 2 diabetes mellitus and hypertension are two major risk factors leading to heart failure and cardiovascular damage. Lowering blood sugar by the sodium-glucose co-transporter 2 inhibitor empagliflozin provides cardiac protection. We established a new rat model that develops both inducible diabetes and genetic hypertension and investigated the effect of empagliflozin treatment to test the hypothesis if empagliflozin will be protective in a heart failure model which is not based on a primary vascular event. The transgenic Tet29 rat model for inducible diabetes was crossed with the mRen27 hypertensive rat to create a novel model for heart failure with two stressors. The diabetic, hypertensive heart failure rat (mRen27/tetO-shIR) were treated with empagliflozin (10 mg/kg/d) or vehicle for 4 weeks. Cardiovascular alterations were monitored by advanced speckle tracking echocardiography, gene expression analysis and immunohistological staining. The novel model with increased blood pressure und higher blood sugar levels had a reduced survival compared to controls. The rats develop heart failure with reduced ejection fraction. Empagliflozin lowered blood sugar levels compared to vehicle treated animals (182.3 ± 10.4 mg/dl vs. 359.4 ± 35.8 mg/dl) but not blood pressure (135.7 ± 10.3 mmHg vs. 128.2 ± 3.8 mmHg). The cardiac function was improved in all three global strains (global longitudinal strain − 8.5 ± 0.5% vs. − 5.5 ± 0.6%, global radial strain 20.4 ± 2.7% vs. 8.8 ± 1.1%, global circumferential strain − 11.0 ± 0.7% vs. − 7.6 ± 0.8%) and by increased ejection fraction (42.8 ± 4.0% vs. 28.2 ± 3.0%). In addition, infiltration of macrophages was decreased by treatment (22.4 ± 1.7 vs. 32.3 ± 2.3 per field of view), despite mortality was not improved. Empagliflozin showed beneficial effects on cardiovascular dysfunction. In this novel rat model of combined hypertension and diabetes, the improvement in systolic and diastolic function was not secondary to a reduction in left ventricular mass or through modulation of the afterload, since blood pressure was not changed. The mRen27/tetO-shIR strain should provide utility in separating blood sugar from blood pressure-related treatment effects.

## Introduction

Systolic blood pressure, smoking and glucose concentrations are the three main risk factors for cardiovascular disease in that order^1^. Type 2 diabetes mellitus combined with hypertension is associated with an enhanced risk, demonstrating the catastrophic combination of these two risk factors^2^. Indeed, hypertension is present in over two-thirds of type 2 diabetes mellitus patients. Hypertension and type 2 diabetes mellitus together meld in heart failure. The prognosis is especially poor in type 2 diabetes mellitus patients because of cardiovascular end organ damage, leading to myocardial fibrosis and diastolic dysfunction^3^. Patients suffering from type 2 diabetes mellitus exhibit subclinical cardiac dysfunction and renal impairment. However, some glucose-lowering therapies actually increase hospital admissions^4,5^. It is known that lowering blood pressure has a dramatic effect on cardiovascular endpoints at all ages. In type 2 diabetes mellitus, the results are less clear^6^. A new class of oral anti-diabetic drugs, sodium-glucose co-transporter 2 (SGLT2) inhibitors, lowers blood glucose in an insulin-independent manner, by inhibiting renal glucose reabsorption in the proximal tubule. The recent Empagliflozin Cardiovascular Outcome Event Trial in type 2 diabetes (EMPA-REG OUTCOME) documented the impressive benefits obtained with empagliflozin (empa)^7^. Empa reduced the primary composite outcome of cardiovascular death, non-fatal myocardial infarction or non-fatal stroke (3-point major adverse cardiovascular events), hospitalization for heart failure, and overall mortality compared with placebo^7^. Myocardial infarction and stroke remained unchanged with therapy, suggesting effects not related to thrombosis^8^. The fact that SGLT2 inhibitors reduce cardiovascular events by reducing primarily heart failure has appeal^9^. New ESC guidelines on chronic coronary syndrome favor SGLT2 inhibitors as primary medication in untreated patients with diabetes before inducing metformin^10^. However, metformin was the primary medication before applying empa in the clinical studies. The question now is whether empa is also cardio protective if administered as the only antidiabetic agent? Several other questions remained unanswered, such as the mechanism of action. An animal model of both hypertension and diabetes might have more utility than either condition alone. We generated a novel rodent model for heart failure by mating rats with type 2 diabetes and hypertension^11,12^. Heart-failure is induced by two stressors, hypertension and diabetes, with state-of-the art technology^13,14^.

The transgenic Tet29 rat model in which the insulin receptor is ubiquitously knocked down via doxycycline (dox)-induced RNA interference (small hairpin RNA), leading to insulin resistance and type 2 diabetes mellitus^11^ is crossed to the mRen27 rat. Transgenic rats carrying the murine Ren27 gene represent a monogenetic model of hypertension induced end organ damage characterized by low plasma renin and high extra renal expression of the transgene^12^. It remains an unanswered question from the clinical trials whether the positive effects of SGLT2 inhibitors on heart failure occur predominantly in heart failure with a preserved ejection fraction (HFpEF) or heart failure with a reduced ejection fraction (HFrEF).

## Methods

### Animal studies

All animal experiments were performed according to national and international guidelines and were approved by local authorities (State Office of Health and Social Affairs Berlin). We used 18 weeks old and body weight-matched male rats to characterize a new rat model for hypertensive and diabetic heart failure. For this purpose, Sprague Dawley healthy control rats (SD; n = 5), diabetic rats (tetO-shIR; n = 10)^11^, hypertensive rats (mRen27; n = 9)^12^, and crossbred diabetic and hypertensive rats (mRen27/tetO-shIR; n = 5) were monitored for 9 weeks. All animals were bred at the Max-Delbrück Center for Molecular Medicine Berlin, Germany. Type 2 diabetes mellitus in the tetO-shIR and mRen27/tetO-shIR rats was induced by oral doxycycline (dox) application. TetO-shIR and mRen27/tetO-shIR rats received 2 mg/kg/d dox via drinking water until they were hyperglycemic with blood glucose level of between 300 and 400 mg/dL. Afterwards, 0.5 mg/kg/d dox was given to maintain the blood glucose level at that level over the entire period of 9 weeks. SD and mRen27 received water. After 8 weeks, blood pressure was monitored by tail cuff measurements and echocardiography was done. Blood glucose was measured manually with the Accu-Chek Aviva blood glucose meter (Roche, Germany) once a week. For the investigation of the effects of empa, 18 weeks old, age-matched and body weight-matched male mRen27/tetO-shIR rats were used. Type 2 diabetes mellitus was induced by dox as described above for 5 weeks. Afterwards, rats were randomly assigned to 2 experimental groups, vehicle-treated (water; n = 13) or treated with 10 mg/kg/d empa in the drinking water (n = 14). The treatment period was 4 weeks. At the end of the study rats were sacrificed by decapitation with prior anesthesia using isoflurane or due to predefined stopping criteria according with the European law for animal protection. Serum brain natriuretic peptide (BNP) was quantified according to the assay manual provided by the manufacturer (Abcam). The experimental unit is described by one single animal.

### Echocardiography

As previously described^15^, transthoracic echocardiography was performed under anesthesia (induction in a chamber with 2%, continuation with 1–1.5% isoflurane) on a tiltable, heated platform to maintain 37 °C body temperature, executed by a single examiner blinded to the treatment groups (n = 6–10 rats per group). Chest hair was gently removed using a clipping machine and depilatory cream. A VisualSonics VEVO 2,100 imaging system with a 21 MHz transducer (MS250) mounted on an integrated rail system was used, simultaneously recording an electrocardiogram. M-mode and B-mode images were obtained in parasternal short and long axes views for measurement of diastolic left ventricular (LV) wall thickness (IVSd, LVPWd) and LV systolic function (EF, FS), respectively. Images were acquired and stored for offline analysis. Offline myocardial strain imaging analysis was performed by an investigator unaware of treatments using VisualSonics Vevo Strain software version 2.2.0 (Toronto, Canada) with a speckle tracking algorithm. B-Mode cine loops were checked for image quality with regard to differentiation of wall borders and absence of artefacts. Strain analysis was performed on three consecutive cardiac cycles. The endocardium of the LV was traced manually in parasternal short- and long-axis view in end-diastole. The epicardium was automatically traced by the software, checked and manually adjusted if necessary for maximum tracking accuracy. Global strain values were obtained from the average of the six segments of the LV. Since echocardiography was performed under anesthesia due to animal welfare aspects and could therefore represent a limitation of this study, special attention was paid to a uniform procedure in the execution and evaluation of the data.

### mRNA isolation and real-time RT-PCR

Total mRNA was isolated from the left ventricle of the heart (n = 5 per group) using QIAzol lysis reagent and Qiagen RNeasy mini kit (Qiagen) with on-column deoxyribonuclease I step (Qiagen) according to manufacturer’s protocol as previously described^16^. 2 μg of mRNA was reverse transcribed into cDNA using High Capacity cDNA Reverse Transcription Kit (Applied Biosystems). Real-time polymerase chain reaction (RT-PCR) was detected on ABI 7500 Fast Sequence Detection System (Applied Biosystems) and analyzed by 7500 Fast System Software (Applied Biosystems). Primers and probes (Supplementary table 1) were designed with PrimerExpress 3.0 (Applied Biosystems) and synthesized by Biotez (Germany). Analysis of target mRNA expression was performed with RT-PCR using the relative standard curve method. The expression level of the target genes was normalized by the expression of the *18S* housekeeping gene. Samples were run in triplicates and mean was used for further calculations. The arbitrary units reflect the ratio of the target mRNA concentration divided by the concentration of *18S* of the same sample.

### Blood pressure and blood glucose measurement

Blood pressure measurement was performed by non-invasive tail cuff (CODA High Throughput System, Kent Scientific Corp., Torrington, USA). Blood glucose was measured by taking capillary blood droplets from tail puncture (Accu-Chek Aviva blood glucose meter, Roche, Mannheim, DEU). The measurements are carried out by animal keepers with decades of experience in a quiet environment to keep the stress for the animals as low as possible.

### Immunohistochemistry

Paraffin-embedded hearts (vehicle n = 6, empa n = 8) were cut 4–6 μm thick and stained. Immunohistological techniques were performed as previously described^17^. Heart sections were incubated with primary antibodies against rat macrophages (ED1; #MCA341R, Bio-Rad), fibronectin (FN; #ab23751, abcam), and collagen I (Col I; #131001, SouthernBiotech). Vectashield mounting medium with DAPI (#H-1200, Vector Laboratories) was used to stain nuclei. Semi-quantitative analyses of immunohistochemistry and histology were conducted without knowledge of the specific treatment and performed using Pannoramic MIDI II slide scanner (3D Histech, Hungary), CaseViewer and Image J software. For the histological and cardiomyocyte analysis, 5 hearts of each group were examined. Picking out cardiac areas for immunohistology were standardized and uniformly distributed over the entire cross-section. Ten to fifteen different areas of each heart were analyzed. For the determination of the perivascular fibrosis, all vessels in the left ventricle were evaluated.

### Statistics

Normal distribution was assessed by Kolmogorov–Smirnov test. Group differences were analyzed by two-tailed unpaired *t*-test, Mann–Whitney *U* test, one-way ANOVA with Tukey post hoc test or two-way ANOVA with Bonferroni post hoc test, as appropriate. Survival was examined using a Kaplan–Meier analysis. The ROUT method was performed for outlier identification with an average false discovery rate less than 1%. A value of *p* < 0.05 was considered statistically significant. All data are presented as means ± SEM. Numbers of evaluated animals are given in the method description.

### Ethics approval

Local authorities approved the studies (State Office of Health and Social Affairs Berlin, Germany), predefined stopping criteria according with the European law for animal protection were included.

## Results

**Figure 1:**
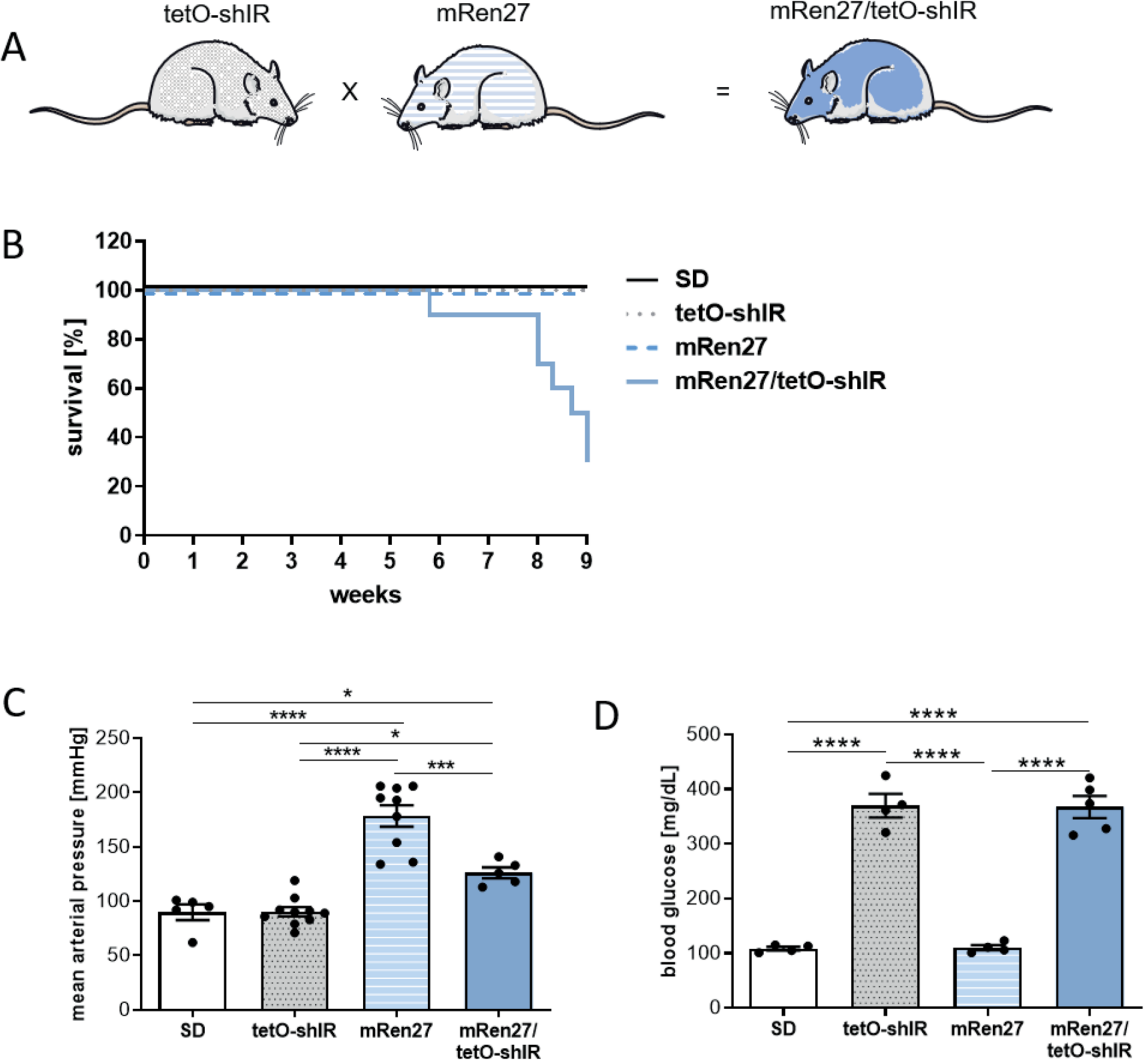
Characteristic of the new animal model. (**A**) Rat model for hypertensive and diabetic heart failure by breeding a hypertensive rat (mRen27), with an inducible diabetic rat (tetO-shIR) resulting in the hypertensive diabetic rat (mRen27/tetO-shIR). (**B**) Kaplan–Meier survival analysis of SD, diabetic (tetO-shIR), hypertensive (mRen27) and hypertensive diabetic rats (mRen27/tetO-shIR). At week 9, only 30% of hypertensive diabetic rats (mRen27/tetO-shIR) survived. Survival curves are statistically different by the log-rank test (*p* < 0.0001). No SD, diabetic (tetO-shIR) and hypertensive (mRen27) rats died before end of study. (**C**) Tail-cuff mean arterial pressure (MAP) was increased in mRen27 rats compared to SD, tetO-shIR and mRen27/tetO-shIR rats. The hypertensive diabetic rats (mRen27/tetO-shIR) showed significantly elevated MAP compared to SD and tetO-shIR rats. SD and tetO-shIR rats were normotensive. (**D**) Blood glucose level were significantly higher in tetO-shIR and mRen27/tetO-shIR rats compared to SD and mRen27 rats. 1-way ANOVA, *p < 0.05, ***p < 0.001, ****p < 0.0001; data expressed as mean ± SEM.

Hypertension and diabetes share many features and often occur together. We therefore bred a hypertensive rat model, the mRen27 rat, with an inducible type 2 diabetes mellitus rat model to generate the mRen27/tetO-shIR rat (Fig. 1A). Hypertensive mRen27 rats and induced diabetic tetO-shIR rats exhibited no mortality in the investigated period. However, 9 weeks of combined hypertension and type 2 diabetes mellitus in mRen27/tetO-shIR rats reduced survival by 30% (Fig. 1B). The mRen27 rats had high mean arterial blood pressure (178 ± 10 mmHg), whereas the diabetic rats (90 ± 4 mmHg) and SD rats (90 ± 7 mmHg) were normotensive. The hypertensive diabetic rats (126 ± 5 mmHg) had lower blood pressures compared to the hypertensive mRen27 rat; however, the values were still significantly higher compared to the normotensive SD rats (Fig. 1C). After induction by doxycycline, type 2 diabetes mellitus (370.0 ± 21.4 mg/dl) and hypertensive, type 2 diabetes mellitus rats (367.6 ± 20.1 mg/dl) exhibited higher blood glucose levels, whereas blood glucose levels in the hypertensive (110.3 ± 4.7 mg/dl) and SD controls (108.5 ± 3.3 mg/dl) were within normal limits (Fig. 1D).

**Figure 2:**
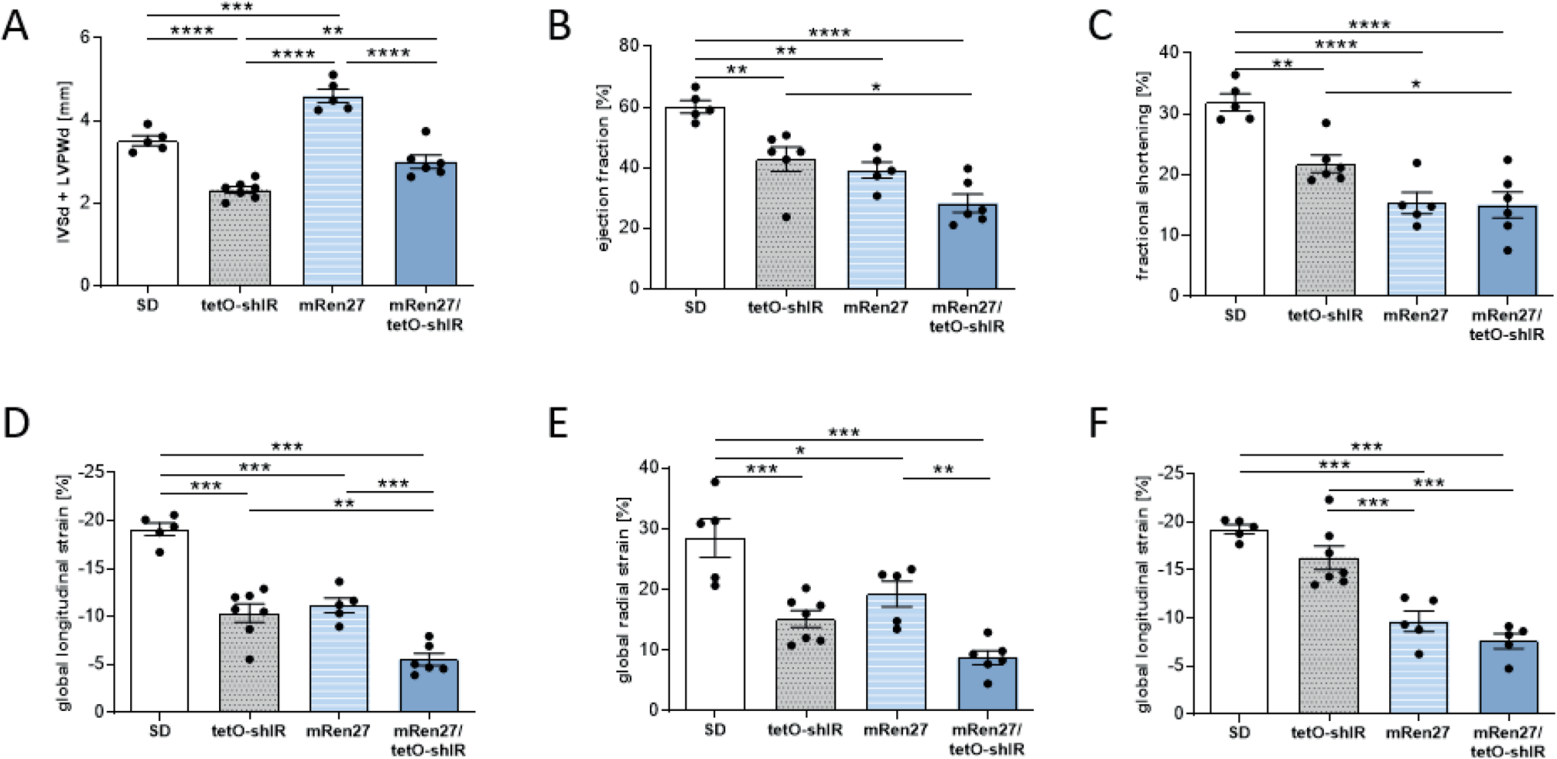
Functional analysis by Speckle Tracking Echocardiography. (**A**) Hypertensive rats (mRen27) showed an increased wall thickness (IVSd + LVPWd) compared to SD, tetO-shIR and mRen27/tetO-shIR rats. Diabetic (tetO-shIR) and hypertensive diabetic rats (mRen27/tetO-shIR) had no thickened cardiac walls. (**B**) Ejection fraction and (**C**) fractional shortening were significantly reduced in tetO-shIR, mRen27 and mRen27/tetO-shIR rats compared to SD. (**D**) Global longitudinal strain was significantly reduced in tetO-shIR, mRen27 and mRen27/tetO-shIR rats compared to SD, as were (**E**) global radial strain and (**F**) global circumferential strain. 1-way ANOVA, *p < 0.05, **p < 0.01, ***p < 0.001, ****p < 0.0001; data expressed as mean ± SEM.

The tetO-shIR rats also had hyperinsulinemia and increased C-peptide levels (data not shown). Echocardiographic assessment of the different models revealed a higher wall thickness (Fig. 2A) calculated by thickness of septum (IVSd) and posterior wall of the LV (LVPWd) in hypertensive mRen27 rats (4.6 ± 0.2 mm) while SD (3.5 ± 0.1 mm), diabetic (2.3 ± 0.1 mm), and hypertensive diabetic rats (3.0 ± 0.2 mm) had no thickened cardiac walls (Fig. 2A). The ejection fraction (Fig. 2B) and fractional shortening (Fig. 2C) values were slightly, but significantly reduced in both diabetic (EF 42.8 ± 4.0%, FS 21.8 ± 1.4%) and hypersensitive rats (EF 39.2 ± 2.7%, FS 15.4 ± 1.8%) compared to SD rats (EF 60.1 ± 2.1%, FS 31.9 ± 1.4%). Systolic heart function was further diminished in the hypertensive type 2 diabetes mellitus rats (EF 28.2 ± 3.0%, FS 15.0 ± 2.1%).

Two-dimensional speckle tracking echocardiography-derived measurement of LV strain and strain rate was performed to examine LV myocardial performance. Global longitudinal strain (GLS, Fig. 2D), global radial strain (GRS, Fig. 2E), and global circumferential strain (GCS, Fig. 2F) were significantly decreased in diabetic (GLS − 10.4 ± 1.0%, GRS 15.2 ± 1.4%, GCS − 16.3 ± 1.2%), hypersensitive (GLS − 11.2 ± 0.8%, GRS 19.3 ± 2.1%, GCS − 9.7 ± 1.1%) and hypertensive diabetic rats (GLS − 5.5 ± 0.6%, GRS 8.8 ± 1.1%, GCS − 7.6 ± 0.8%) compared with SD rats (GLS − 19.1 ± 0.7%, GRS 28.5 ± 3.2%, GCS − 19.3 ± 0.5%). Similar results were observed for the respective global strain rates (data not shown).

**Figure 3:**
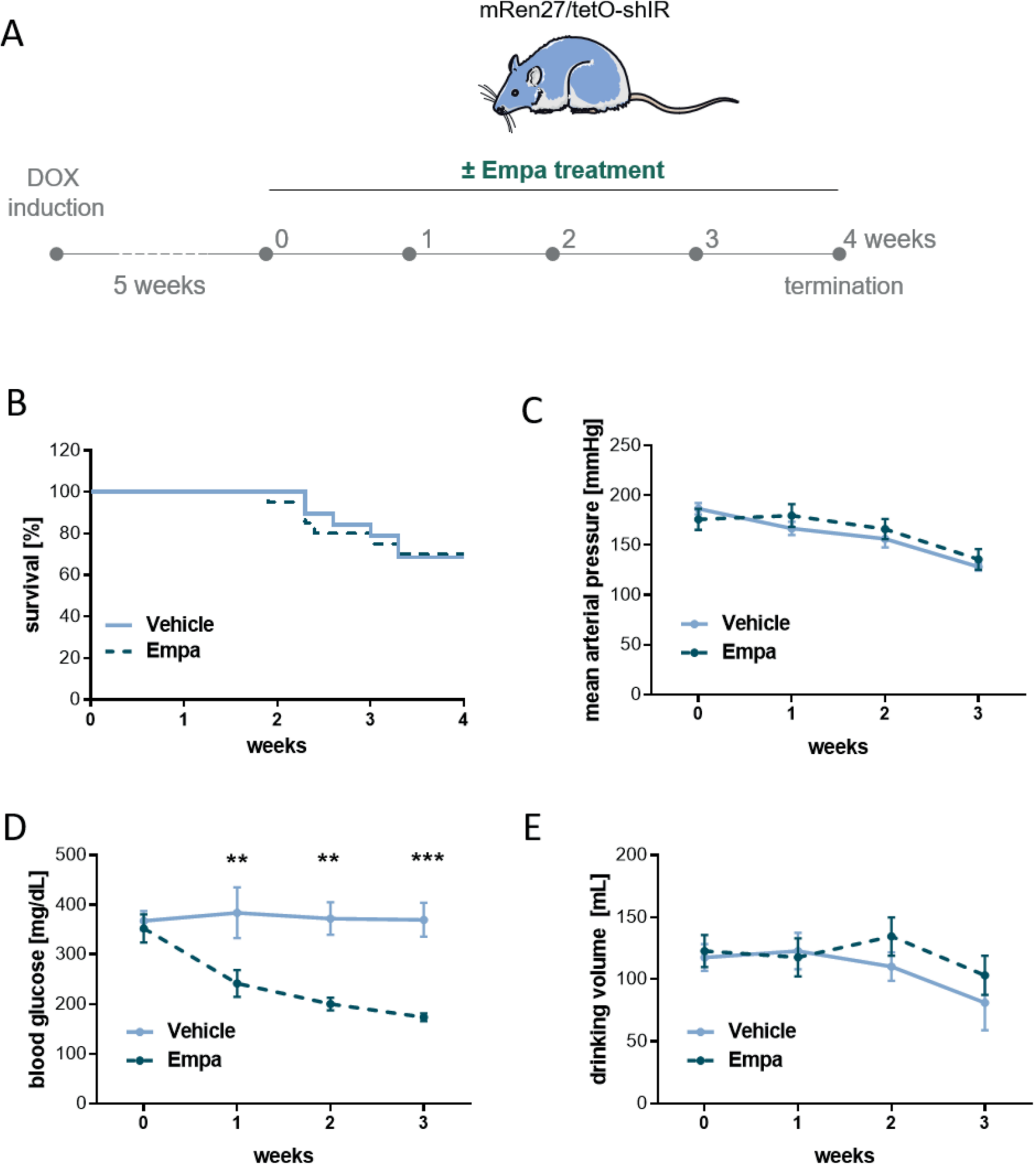
Study design and outcome after empa treatment. (**A**) Experimental flow chart of EMPA study design. (**B**) Kaplan–Meier survival analysis showed no effect of empa treatment on survival and no effect on (**C**) mean arterial blood pressure. (**D**) Blood glucose level were significantly reduced by empa during the treatment period. (**E**) Empa treatment had no effect on the drinking volume. 2-way ANOVA, **p < 0.01, ***p < 0.001; data expressed as mean ± SEM.

According our empa study design (Fig. 3A), we investigated the effects of empa on the cardiac target-organ damage in the hypertensive diabetic rats (mRen27/tetO-shIR). The survival rates (Fig. 3B) during the 4 weeks of treatment were unchanged between the empa (70.0%) and vehicle group (68.4%). Treatment of the hypertensive diabetic rats by empa had no effect on the blood pressure (Fig. 3C). Empa treatment led to significant lower blood glucose level (Fig. 3D). Glucose level decreased from 359.4 ± 35.8 mg/dl in vehicle treated rats to 182.3 ± 10.4 mg/dl in empa treated ones. The drinking volumes of empa treated rats were unaltered compared to the vehicle group (Fig. 3E).

**Figure 4:**
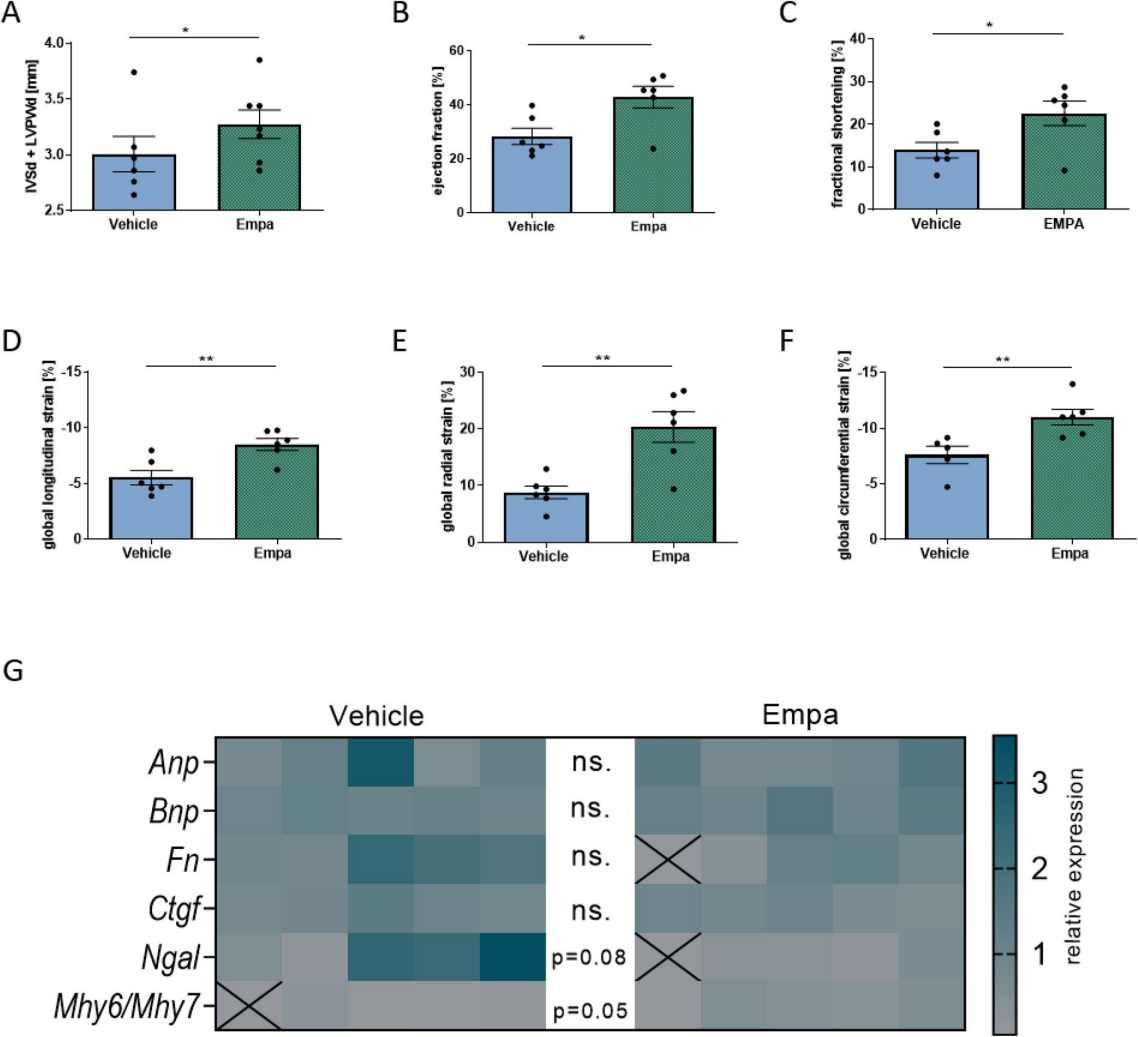
Speckle Trackle Echocardiography and gene expression analysis after empa treatment. (**A**) No effects of empa treatment on wall thickness could be detected. (**B**) Ejection fraction and (**C**) fractional shortening were significantly increased by empa. (**D**) Global longitudinal strain was significantly elevated by empa treatment, as were (**E**) global radial strain and (**F**) global circumferential. (**G**) Heatmap of gene expression values in relation to *18S* housekeeper are shown for every single animal. Relative gene expression is illustrated in corresponding color. Statistical outliers are marked with X. There is no change in cardiac atrial natriuretic peptide (*Anp*), brain natriuretic peptide (*Bnp*), fibronectin (*Fn*) or connective tissue growth factor (*Ctgf*) mRNA expression due to empa treatment. The ratio of alpha (*Mhy6*) and beta myosin heavy chain (*Mhy7*) mRNA expression is increased by treatment. Students t-test, *p < 0.05 **p < 0.01; data expressed as mean ± SEM.

We then assessed the effect of empa on cardiac function by echocardiography. No effects of empa treatment on wall thickness could be detected (Fig. 4A). Empa significantly increased the ejection fraction (42.8 ± 4.0%) and fractional shortening (22.6 ± 2.9%) compared to vehicle treated hypertensive diabetic rats (Fig. 4B-C). Moreover, empa has a positive effect on all 3 global strains. Global longitudinal (− 8.5 ± 0.5%), radial (20.4 ± 2.7%) and circumferential (− 11.0 ± 0.7%) strains were significantly enhanced by empa treatment compared to the vehicle group (Fig. 4D-F). Similar results were observed for the global longitudinal and radial strain rates (data not shown).

**Figure 5:**
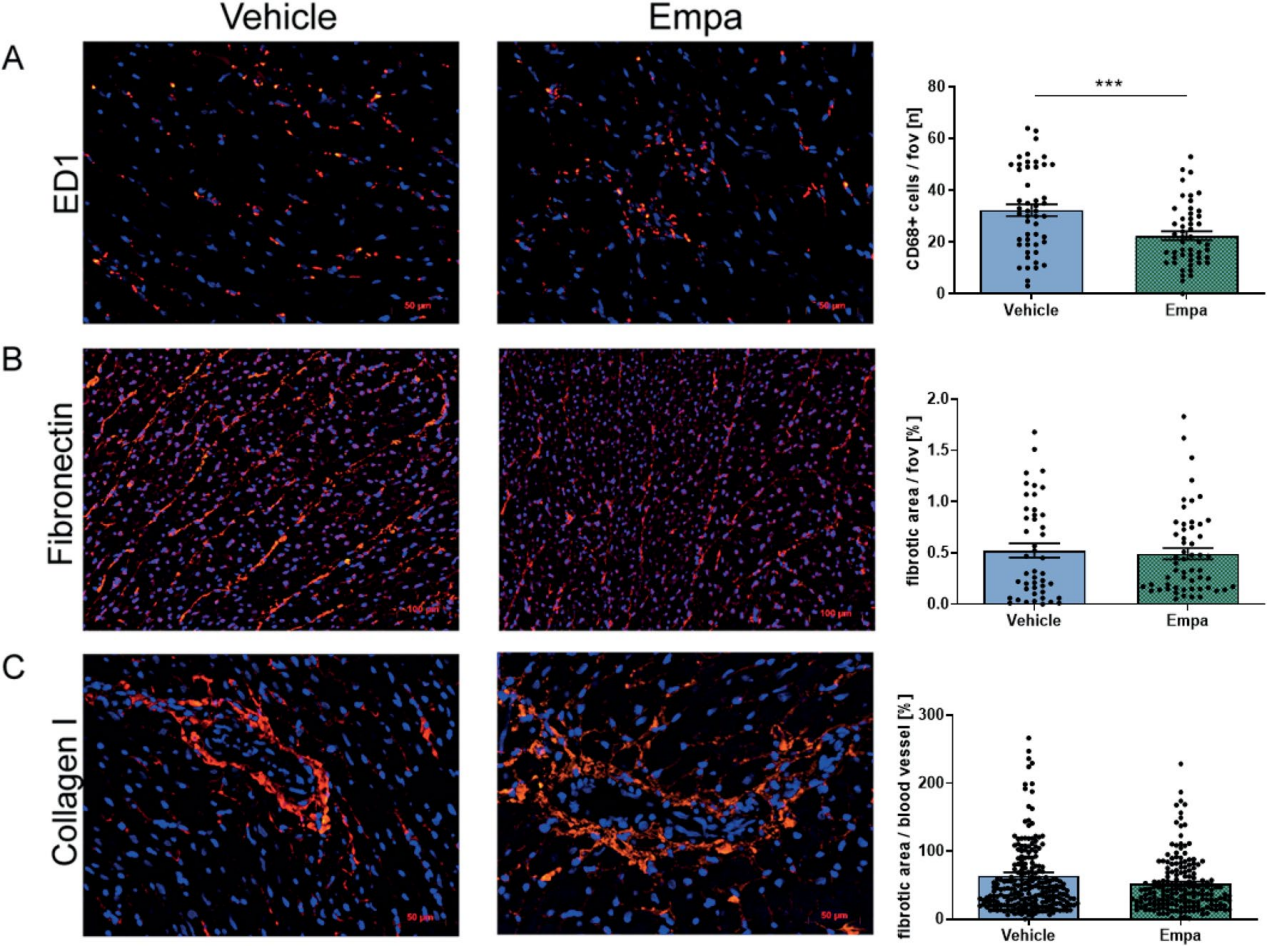
Protein expression after empa treatment. (**A**) Representative image of ED-1 IHC staining for macrophages in the heart from empa and vehicle-treated rats. The number of macrophages was decreased in the heart of empa treated rats. (**B**) Representative image of fibronectin IHC staining in the hearts from empa and vehicle-treated rats. Interstitial cardiac fibrosis was estimated as fibronectin-positive area per view field. Heart sections of empa and vehicle-treated rats showed no differences in cardiac interstitial fibrosis. (**C**) Representative image of collagen I IHC staining in the hearts from empa and vehicle-treated rats. Perivascular fibrosis area was normalized to vessel media cross-sectional area. Heart sections of empa and vehicle-treated rats showed no differences in cardiac perivascular fibrosis. Students t-test, *** *p* < 0.001; data expressed as mean ± SEM.

The mRNA expression level of the cardiac marker *Anp* (1.37 ± 0.45 vs. 1.17 ± 0.20)*, Bnp* (1.10 ± 0.05 vs. 1.26 ± 0.12)*, Fn* (1.57 ± 0.31 vs. 0.93 ± 0.19) *and Ctgf* (0.99 ± 0.12 vs. 0.82 ± 0.08) were unchanged. Only an increase in the ratio of *Myh6/Mhy7* mRNA expression in empa treated rats (0.14 ± 0.05) compared to vehicle treated animals (0.40 ± 0.09) were detectable (Fig. 4G). Additionally, we analyzed circulating BNP levels that were also unaltered by empa treatment (data not shown). Immunohistochemical staining for cardiac macrophage infiltration (ED1), interstitial (fibronectin) and perivascular fibrosis (collagen I) was also investigated. Macrophage infiltration (Fig. 5A) was decreased in the cardiac tissue in the empa treated rats (22.4 ± 1.7 per fov) compared to vehicle treated (32.3 ± 2.3 per fov) whereas interstitial (Fig. 5B) and perivascular (Fig. 5C) fibrosis was not affected by empa treatment.

## Discussion

We investigated the combined effects of hypertension and hyperglycemia on end-organ damage, focusing on the heart. We used a well-established transgenic rat model, overexpressing the mouse salivary-gland renin gene. This model is based on increased angiotensin II production resulting in a rightward shift in the pressure natriuresis curve^18^. We crossed these animals with a unique tetracycline-inducible type 2 diabetes mellitus technique where insulin resistance results from downregulation of the insulin receptor^11^. Almost two third of the hypertensive type 2 diabetes mellitus rats died between week 6 and 9 in our experiment, underscoring the lethality of hypertension plus hyperglycemia-induced heart failure. Surviving rats that could be measured had a marked reduction of systolic function and the worst global longitudinal strain, radial strain, and circumferential strain, thus we have introduced a novel model of two step heart failure (HF). In most rodent HF models, HF develops gradually over time due to a single stimulus. However, in human HF multiple factors are involved and trigger decompensation episodes. Among these factors, arterial hypertension is a prevalent trigger for decompensation episodes. We succeeded in establishing a novel HF rat model, which combines two incumbent protocols to achieve both the development of HF and the possibility to add a second stimulus to induce (HFrEF).

Hypertensive diabetic rats as a model have been investigated earlier. Iams and Wexler produced diabetes with alloxan in spontaneously hypertensive rats (SHR) in 1977^19^. Mortality was high and animals became cachectic and moribund, probably related to acute metabolic disturbances rather than target-organ damage. The authors did not report data regarding blood pressure. Streptozotocin has also been commonly given to SHR. Hori et al.^20^ reported on basement membrane changes in various tissue but also did not report on blood pressure-related effects. Techniques designed to destroy islet beta cells are more akin to type 1 diabetes mellitus rather than type 2 diabetes mellitus. Rats with type 2 diabetes mellitus present a significant challenge. Most rodent models are genetically or chemically modified to produce diabetes. Commonly high-fat-feeding is required, the length of time to develop diabetes is quite long and the phenotype is variable. The Nile rat may be an exception^21^. Nile rats develop diabetes within 8–10 weeks with high-carbohydrate diet similar to humans, and are protected by high-fat, low-glycemic load intake^21^. That model, with less carefully defined insulin levels, proved unpractical for our purposes. Thus, we selected the tetO-shIR rat, which allowed us to avoid additional dietary manipulations. Another unique advantage is that the type 2 diabetes mellitus can be turned on and off with tetracycline.

We tested the hypothesis if empagliflozin will be protective in a heart failure model which is not based on a primary vascular event. Controls were mRen27/tetO-shIR given vehicle. Empa lowered plasma glucose levels from 400 mg/dL to under 200 mg/dL, a remarkable reduction based on increased glucose excretion alone. Blood pressure was not changed by this treatment. Nonetheless, empa improved cardiac function by increasing ejection fraction and fractional shortening compared to vehicle-treated controls. Moreover, empa has a positive effect on all 3 global strains. Global longitudinal, radial, and circumferential strains were significantly enhanced by empa treatment compared to the vehicle group. Since no cardiac expression of SGLT2 receptors has been described, it is difficult to postulate any direct effect of the drug on the myocardium per se. However, it is possible that empagliflozin binds to myocardial receptors or substrates other than SGLT2. In our model the improvement in systolic and diastolic function was not secondary to a reduction in left ventricular mass or through modification of the afterload since blood pressure was not changed.

Empa has been previously studied in other rodent models of hypertension and diabetes. Younis et al. studied the Cohen Rosenthal diabetic hypertensive rat^22^. This model is an SHR cross with the Cohen diabetic rat. Unlike in our model empa lowered blood pressure by 15 mmHg and also reduced left ventricular mass and systolic dilatation. Steven et al. also reported favorable results in the Zucker diabetic fatty rat^23^. They found that endothelial function was improved and inflammatory markers were reduced. Similarly, Zhou and Wu observed that empa treatment in streptozotocin-induced diabetes reduced serum glucose, while improving cardiac function and lowering expression of glucose-regulated protein-78 and enhancer-binding protein homologous protein^24^. Thus, it is obvious that several, putative mechanisms underlying SGLT2 inhibitor-associated cardiovascular benefits occur as in patients as in rodents.

Whether or not the mechanism(s) involved in glycemic control and cardiovascular risk reduction are dissociated and/or follow a different dose–response-curve is unclear. Due to the profound and early effects of empa on HF in patients and the fact that they were not due to improvement in atherothrombotic complications (myocardial infarction and stroke remained unchanged), it is difficult to ascribe all these salubrious effects solely to serum glucose reduction^25^.

Ascribing the favorable effects to altered ventricular loading conditions secondary to a reduction in preload primarily due to the diuretic and natriuretic effects is an alternative mechanism. SGLT2 inhibition in the proximal tubule leads to natriuresis and glucosuria, and the ensuing osmotic diuresis may be favorable, particularly in the diabetic and hypertensive heart, which functions on a steep Frank-Starling curve. We observed no effects of empa on drinking volume, although we could not monitor daily urinary output. This must be kept in mind when comparing the circulating volumes. In patients with heart failure, a differential effect in the regulation of interstitial fluid (versus intravascular volume) may be particularly important, in many of whom there is intravascular contraction, often exacerbated by diuresis. The ability to selectively reduce interstitial fluid may be a unique feature of SGLT2 inhibitors. Other diuretic SGLT2 inhibitors may directly inhibit the Na+/H+ exchanger 1 isoform in the myocardium^26,27^, reduced cytoplasmic sodium and calcium levels, while increasing mitochondrial calcium levels.

SGLT2 inhibitors have shown to optimize cardiac energy metabolism and subsequently improve myocardial energetics and substrate efficiency. In a recent study empagliflozin increased ketone consumption, reduced cardiac lactate production and glucose consumption after myocardial infarction in pigs^28^. Alternatively empa inhibits the Na+/H+ exchanger, lowering cytosolic sodium and calcium levels and increasing mitochondrial calcium concentrations^27^.

Taken together, our observations suggest the potential of empagliflozin to favorably promote left ventricular reverse remodeling and improve diastolic function in patients with type 2 diabetes and hypertension induced cardiovascular disease. A trial to test the effects of empa on the heart in patients is underway^29^. There is a reason to be optimistic since only SGLT2 inhibitors and glucagon-like peptide 1 agonists have been shown to any favorable effects on reducing mortality in type 2 diabetes mellitus patients^30^.

## Conclusion

The EMPA-REG OUTCOME trial documented impressive empagliflozin benefits on heart failure in diabetic patients with cardiovascular disease. A cardiovascular protective effect was more primary on preventing systolic heart failure morbidity and mortality than on preventing vascular events such as stroke and acute myocardial infarction. New ESC guidelines on chronic coronary syndromes favor SGLT2 inhibitors as primary medication in untreated patients with diabetes before introducing metformin. The key question of this study was if empagliflozin will be protective in a heart failure model which is not induced by a primary vascular event, such as myocardial infarction, leading to heart failure with reduced ejection fraction. Therefore, we developed an innovative rodent model for HFpEF which progresses to heart failure with mildly reduced ejection fraction. Empagliflozin ameliorated cardiovascular dysfunction in this model. The beneficial effect was not secondary to a reduction in LV mass or through modulation of the blood pressure. In future, SGLT2 inhibitors will become an important primary vascular preventive pillar next to lipid lowering and platelet inhibition independent of the diagnosis diabetes. Future studies will determine the exact mode of action and the patient groups with chronic coronary syndrome and heart failure who will benefit most.

## Supplementary information

Supplementary Table 1.

## Data Availability

The datasets used and analysed during the current study are available from the corresponding author on reasonable request.

## Abbreviations

Anp: Atrial natriuretic peptide (gene)
Bnp: Brain natriuretic peptide (gene)
BNP: Brain natriuretic peptide (protein)
Col1: Collagen type 1
Ctgf: Connective tissue growth factor (gene)
DOX: Doxycycline
ED1: CD68^+^ macrophages
EF: Ejection fraction
empa: Empagliflozin
FN: Fibronectin
FS: Fractional shortening
GCS: Global circumferential strain
GLS: Global longitudinal strain
GRS: Global radial strain
HF: Heart failure
HFpEF: Heart failure with preserved ejection fraction
HFrEF: Heart failure with reduced ejection fraction
IVSd: Interventricular septum, diastole
LV: Left ventricular
LVPWd: Left ventricular posterior wall, diastole
MAP: Mean arterial blood pressure
mRen27: Hypertensive rat model
mRen27/tetO-shIR: Hypertensive, diabetic heart failure rat model
mRNA: Messenger ribonucleic acid
Myh6/7: Myosin heavy chain 6/7
RT-PCR: Real-time polymerase chain reaction
SD: Sprague Dawley
SEM: Standard error of the mean
SGLT2: Sodium-glucose co-transporter 2
SHR: Spontaneously hypertensive rats
Tet29: Diabetic rat model (tetO-shIR
