# Supplementary material for: Effects of empagliflozin and target-organ damage in a novel rodent model of heart failure induced by combined hypertension and diabetes
Source: Sci Rep. 2020 Aug 20;10:14061. doi: 10.1038/s41598-020-70708-5 (PMC7441148; doi:10.1038/s41598-020-70708-5)
Supplement: Supplementary file 1 — Supplementary Table 1. [file 41598_2020_70708_MOESM1_ESM.doc]

**Effects of empagliflozin and target-organ damage in a novel rodent model of heart failure induced by combined hypertension and diabetes**

Kristin Kräker*^1,2,3,4,5^ Florian Herse*^1,2,3,5^; Michaela Golic ^1,2,3,4,5^; Nadine Reichhart^6^; Sergio Crespo-Garcia^6,7^; Olaf Strauß^6^; Jana Grune^4,8,9,10^; Ulrich Kintscher^4,9^; Manal Ebrahim^11^; Michael Bader^2,3,4,5^; Natalia Alenina^3,4^; Arnd Heuser^3^; Friedrich C Luft^1,3,4^; Dominik N Müller^1,2,3,4,5^; Ralf Dechend*^1,2,5,12^; Nadine Haase*^1,2,3,4,5^

^1^Experimental and Clinical Research Center, a joint cooperation between the Max-Delbrück Center for Molecular Medicine in the Helmholtz Association and the Charité Medical Faculty, corporate member of Freie Universität Berlin, Humboldt-Universität zu Berlin, and Berlin Institute of Health, Berlin, Germany

^2^Berlin Institute of Health (BIH), Berlin, Germany

^3^Max-Delbrück Center for Molecular Medicine in the Helmholtz Association, Berlin, Germany

^4^DZHK (German Centre for Cardiovascular Research), partner site Berlin, Germany

^5^Charité – Universitätsmedizin Berlin, corporate member of Freie Universität Berlin, Humboldt-Universität zu Berlin, and Berlin Institute of Health, Berlin, Germany

^6^Experimental Ophthalmology, Department of Ophthalmology, Charité – Universitätsmedizin Berlin, corporate member of Freie Universität Berlin, Humboldt-Universität zu Berlin, and Berlin Institute of Health, Berlin, Germany

^7^Department of Biochemistry, Université de Montréal, Montreal, Canada

^8^Institute of Physiology, Charité - Universitätsmedizin Berlin, corporate member of Freie Universität Berlin, Humboldt-Universität zu Berlin, and Berlin Institute of Health,Berlin, Germany

^9^Institute of Pharmacology, Center for Cardiovascular Research, Charité - Universitätsmedizin Berlin, corporate member of Freie Universität Berlin, Humboldt-Universität zu Berlin, and Berlin Institute of Health, Berlin, Germany

^10^Center for Systems Biology, Massachusetts General Hospital, Harvard Medical School, Boston, USA

^11^Technische Universität Berlin, Berlin, Germany

^12^HELIOS-Klinikum, Berlin, Germany

* shared first and last authorship

**Total word count** 5791

**Corresponding Author**

Ralf Dechend, MD

Experimental and Clinical Research Center

Lindenberger Weg 80

13125 Berlin, Germany

Tel.: +49-30-450540302, Fax: +49-30-450540944

E-Mail: [ralf.dechend@charite.de](mailto:ralf.dechend@charite.de)

**Online Data Supplement**

Supplementary table 1. Primer sequences used for RT-PCR.

| Gene |  | Sequences (5’→3’) |
| --- | --- | --- |
| *α-Mhc* | for | CGGGAGAACCAGTCCATCCT |
|  | rev | ACACGCTTCGTGTTGACAGTCT |
|  | probe | SYBR |
|  |  |  |
| *β-Mhc* | for | GCCAAGACAGTTCGGAATGATAA |
|  | rev | CCTGTTGCCCCAAAATGG |
|  | probe | SYBR |
|  |  |  |
| *Ctgf* | for | CGCCAACCGCAAGATTG |
|  | rev | CACGGACCCACCGAAGAC |
|  | probe | CACTGCCAAAGATGGTGCACCCTG |
|  |  |  |
| *Anp* | for | CCAGGCCATATTGGAGCAGCAAA |
|  | rev | GCAGGTTCTTGAAATCCATCAGA |
|  | probe | CCCGTATACAGTGCGGTGTCCAACACA |
|  |  |  |
| *Bnp* | for | CAAGCTGCTTTGGGCAGAAG |
|  | rev | AAACAACCTCAGCCCGTCAC |
|  | probe | AGACCGGATCGGCGCAGTCAGTCGCTT |
|  |  |  |
| *18S* | for | ACATCCAAGGAAGGCAGCAG |
|  | rev | TTTTCGTCACTACCTCCCCG |
|  | probe | CGCGCAAATTACCCACTCCCGAC |
